# Review of the recruitment process for a large investigator-initiated trial in early Parkinson’s disease

**DOI:** 10.1186/s13063-022-06052-y

**Published:** 2022-02-14

**Authors:** C. V. M. Verschuur, J. L. Donovan, R. M. A. de Bie

**Affiliations:** 1 Department of Neurology, Amsterdam Neuroscience, Amsterdam UMC, University of Amsterdam, Meibergdreef 9, 1105 AZ Amsterdam, The Netherlands; 2 Bristol Medical School, University of Bristol, Bristol, UK

**Keywords:** Parkinson, Levodopa, LEAP trial, Organization, QuinteT Recruitment Intervention

## Abstract

**Introduction:**

Organizing and executing a large clinical trial is a complex process, and often recruitment targets are not met. We describe the organization of the Levodopa in the Early Parkinson’s disease (LEAP) trial and the results of an external assessment of the recruitment process.

**Methods:**

Several strategies were used to ensure that recruitment for the trial was effective and efficient. We analyzed the patterns in referrals, inclusions, and non-inclusions to investigate whether there were bottlenecks in the referral and inclusion process. For the external assessment of the recruitment process, the QuinteT Recruitment Intervention (QRI-Two) was used retrospectively, focusing on finding possible issues impeding recruitment that are less easily recognized.

**Results:**

Recruitment took 57 months, which was 27 months longer than initially expected. 6.8% of the estimated eligible patients in the Netherlands were included. The number of referrals differed widely between participating centers and regions in the Netherlands, with the region of the principal study center having the most referrals. Reasons of exclusion varied across regions, as in some regions more patients already started, wanted to start, or did not want to start with Parkinson medication compared to other regions.

**Discussion:**

Executing a large, investigator-initiated clinical trial on a limited budget still remains possible by focusing on minimizing administrative and organizational procedures. Our study suggests that centers with closer institutional ties to a principal study center tend to have a higher referral rate. The review of the LEAP trial recruitment strategies and data using the QRI-Two suggested that the variations in referrals and reasons of non-inclusion could indicate the presence of issues related to clinical equipoise, patient eligibility, or study presentation. Integrating a recruitment intervention could have explored issues with study presentation and equipoise that might have increased recruitment efficiency.

**Trial registration:**

ISRCTN ISRCTN30518857. The registration was initiated on 02/08/2011 and finalized on 25/08/2011. Recruitment started on 17/08/2011, after the initiation of public registration.

## Key points

- Executing a large, investigator-initiated clinical trial on a limited budget still remains possible by focusing on minimizing administrative and organizational procedures.
- Our study suggests that centers with closer institutional ties to a principal study center tend to have a higher referral rate.
- A review of the recruitment strategies and data using a QRI-Two indicated that prospectively integrating a recruitment intervention could have provided insights about equipoise, eligibility, and study presentation issues in order to increase recruitment efficiency.

## Background

Before patient recruitment for a trial can be started, a complex and intensive process has preceded: a sound trial plan has to be designed, and a myriad of practical questions have to be answered concerning patient recruitment, execution of assessments, developing a database, organizing trial and data monitoring, and obtaining funds. Unfortunately, recruitment targets are often not met, resulting in an underpowered trial or an extended recruitment period, with only partially answered or unanswered questions, and a waste of resources. In a Cochrane Review, it was estimated that these problems occur in more than 50% of trials [1], and a review of trials funded by two large public funding agencies in the UK showed that only 31% of those trials successfully completed their recruitment target [2]. Hence, there is a need to learn from clinical trials that managed to recruit the desired number of patients and, moreover, succeeded in this on a limited budget.

For the motor symptoms of Parkinson’s disease (PD), levodopa is the mainstay of therapy because it is very efficacious in relieving symptoms and it is inexpensive [3]. From the 1980s until the 2010s, many neurologists tended to delay initiation and timely adjustments of levodopa because of concerns that levodopa could be toxi c[4]—although this was never supported by the results of clinical studies—and that an early start with levodopa would induce more severe motor fluctuations and dyskinesias later [5]. Because it is important to know whether levodopa has a disease-modifying effect in Parkinson’s disease in addition to its symptomatic effect, we initiated the Levodopa in Early Parkinson’s disease (LEAP) trial. The trial design and its results have been published previously [6, 7].

In this paper, we describe the organization of the trial, including strategies to tackle anticipated recruitment and retainment challenges. In addition, the results of an external assessment of the recruitment process are presented.

## Methods

### Trial design

To separate possible disease-modifying effects from the direct symptomatic effect of levodopa, a multi-center, randomized delayed-start, double-blind placebo-controlled trial design was used. Patients with early PD whose functional health did not yet warrant initiation of PD medication were randomized to either 80 weeks of treatment with levodopa/carbidopa or 40 weeks placebo followed by levodopa/carbidopa for 40 weeks. A total of eight study visits were necessary to be able to adequately measure the progression of symptoms. To be able to detect a minimal clinically important difference of 4 points on the Unified Parkinson’s Disease Rating Scale at 80 weeks, 446 patients needed to be included [6].

### Inclusion feasibility

Planning for the trial took place in the beginning of 2010. To complete recruitment in a reasonable timeframe of a few years, we estimated the number of potentially eligible patients in the Netherlands, as this would indicate how many centers were needed to refer patients for the trial. In December 2009, the population in the Netherlands aged between 55 and 75 numbered 3,541,977 people [8]. The incidence of parkinsonism in this age group is 2.67 per 1000 per year [9]. De Lau and colleagues found that 51% of these patients fulfilled the criteria for PD, and of those 61% had visited a neurologist [9]. In the Netherlands, almost exclusively neurologists diagnose PD. Accordingly, we estimated that each year approximately 2942 patients were diagnosed with PD by a neurologist in the Netherlands. In the Comorbidities and Aging in Rehabilitation Patients: the influence on Activities (CARPA) study, which was initiated to assess the long-term course of functional status in newly diagnosed PD patients from outpatient clinics of six non-academic hospitals in the Netherlands, 31.6% did not use symptomatic treatment at the time of inclusion [10]. Based on this study, we anticipated that 930 patients would be eligible for the LEAP trial each year and that, in 30 months, 446 patients of the estimated 2325 available patients with early PD should be included, which is 19.2%. Therefore, all hospitals in the Netherlands—also those with general neurologists, usually less prone to participate in studies—were asked to help recruit patients. After approaching about 90 centers in the Netherlands by telephone and e-mail in 2010, 62 centers signed the “Intention to Participate” Statement (IPS). These centers were spread throughout the Netherlands and consisted of almost all hospitals in the Netherlands. Because in the Netherlands PD care almost exclusively takes place in the setting of the outpatient clinic in a hospital, the trial could potentially recruit patients from almost the whole country. Interestingly, at the time these centers estimated the total number of eligible patients at 793 per year. This would imply an inclusion ratio of 22.5% per year.

To verify whether our estimated inclusion ratio was expected to be realistic, we also examined other relevant studies. As the TEMPO-, ELLDOPA- and ADAGIO-trials all had similar target populations—recruiting patients with early PD in most Western countries, in a randomized RCT using a placebo—these trials could be examined to verify whether the projected inclusion ratios of 19.2 and 22.5% were realistic [3, 11, 12]. Unfortunately, the ADAGIO- and ELLDOPA-trial did not report the number of referred or screened patients prior to inclusion, and in the TEMPO trial, an inclusion rate of 85.4% was reported—very high for any trial—raising the question if there may have been a non-reported preselection of patients. Patients in the TEMPO trial furthermore only gave consent for the 26-week trial, and not yet to the later added open-label extension of the trial, thus making a known inclusion ratio less relevant. Based on the sparse knowledge at hand and the fact that no other trial recruited patients from our target population, we expected that an inclusion time frame of 30 months was reasonable.

### Recruitment and assessment logistics

The trial infrastructure consisted of five regional main study centers strategically spread throughout the Netherlands, of which one was the principal study center. At each of the five centers, a trained research nurse was stationed. When a neurologist diagnosing PD thought a patient was eligible, the trial was briefly introduced, printed trial information was provided, and permission was asked to send contact information to the principal study center. This information consisted of the date of referral, name of the referring neurologist and center, a filled-in checklist with inclusion and exclusion criteria, and the patient’s name, date of birth, address, and phone number.

After the contact information was registered at the principal study center, the research nurse at the patient’s regional main study center was automatically informed by e-mail. Within three working days, the referred patient was contacted by phone to answer possible initial questions of the patient, and an appointment was made to further explain the trial. All procedures—informing the patient, inclusion, randomization, and assessments—were performed by the research nurse at the patient’s local center or home, and supervised by the researcher in the regional main study center. Patients were compensated for travel costs. When an assessment was performed in a local center, it only had a facilitating role in providing an outpatient clinic room, as all necessary equipment was brought by the research nurse. As a result, the time investment for referring neurologists was kept minimal, continuity of research nurses for patients was guaranteed at a high degree, research nurses could focus on the LEAP trial because of the large number of assessments that were scheduled, and personnel costs were low. The referring neurologist remained the treating and main responsible neurologist for the patient during the study.

### Trial awareness and communication

Considerable effort was directed to raising trial awareness before and during the trial. The communication strategies primarily targeted neurologists in all centers that signed the IPS, since these could refer patients for the trial. All communication, management, and administrative tasks were executed by the coordinating research nurse at the principal study center, principal investigator, two PhD students, the research nurses at the regional main study centers, and occasional administrative help from medical students.

#### Before trial initiation

Before starting the trial, the main contact person of a center received a scripted phone call to explain the trial, emphasizing that the results of the trial would have clinical consequences, and that time investment for checking study eligibility and referral was kept to a minimum—having in mind the busy schedule of a general neurologist. Answers to questions were prepared beforehand. Six months before starting recruitment, a kick-off presentation was given to explain the trial in all centers that signed the IPS. During this presentation, it was explained how the trial team thought what would be the least confronting method to introduce the trial by the neurologist—often very soon after diagnosis: first, it was advised to inform the patient that there is a large trial in the Netherlands for patients with early PD investigating when to start with PD medication and that the neurologist thinks the patient could be eligible for the trial. Second, the patient could be asked if they consented that contact information was sent to the principal study center in order for a research nurse to contact the patient and further explain the trial. Furthermore, as the trial team did not budget reimbursements for recruiting neurologists, it was explained how the trial team minimized the time and administrative burden for participating neurologists, and that the main contact person of referring center was included in the LEAP Study Group—and with that in the main article publishing the results of the trial. **I**f it was not possible to plan a presentation, the center was not allowed to refer patients because of an expected lack of commitment.

Finally, a study website (leapamc.nl) was developed by a professional website designer, and accounts at Facebook and Twitter were set up to regularly share trial news. Pocket cards and posters with the inclusion and exclusion criteria and the registration process were handed out for the offices in the outpatient clinics of all referring centers.

#### During the trial

To reraise awareness and receive feedback concerning the logistics of the trial, the main contact person of all referring centers was called on a regular basis, asking them to keep the trial under the attention of his or her colleagues, how we could further accommodate the neurologists at that center, whether there were any issues in relation to the clinical follow-up of included patients, and to again explain how the trial team thought what would be the best way to introduce the trial.

Monthly e-mails were sent targeting all participating neurologists to inform about the trial progress using a dashboard with an overview of the inclusion progress. Each time an important inclusion milestone was met, a newsletter was sent to all referring centers, combined with a large cake, pens, or mugs—all having the LEAP logo clearly visible. Approximately 1 year after the start of enrollment, all centers that actively referred patients were approached for a second presentation to give an update about the trial progress and reraise awareness of the trial. Two times, a presentation was allowed during the yearly congress of the Dutch Movement Disorders Working Group.

#### Patients

To bring the trial to the attention of possibly eligible patients with early PD, a presentation was given at a large patient congress early in the course of the trial, and two lay publications explaining the trial were published in the magazine of the Dutch PD patient association. Before initiation of the trial and during the trial, all research nurses were trained at several teambuilding meetings on the study procedures during the contacts leading up to the inclusion of a patient. During these trainings, instructions were given about the best, neutral ways to present trial information to an eligible patient—having in mind that patients were necessarily asked to participate in a trial at an emotionally difficult moment soon after being diagnosed with PD.

### Medication logistics

Safe manufacturing, packaging, labeling, storing, and shipment of trial medication is highly specialized, and time-consuming and administrative demands concerning accountability are equally demanding—even for levodopa, the most commonly used PD-medication for several decades. The trial team contracted ACE Pharmaceuticals and ACE Apothecary (Zeewolde, the Netherlands, Good Manufacturing Practice accredited) to execute all trial medication-related issues, instead of conventionally contracting the pharmacies of all referring centers or those at the main study centers. Much time and effort were saved for the trial team in this way, and probably made the risk of trial medication-related mistakes smaller. After the digital randomization by a research nurse, an e-mail was automatically generated by the database and sent to ACE Pharmaceuticals and ACE Apothecary. Subsequently, the trial medication was shipped to the patient’s home. This ensured that the principal study center only had one professional partner responsible for all trial medication-related issues.

### Medical ethical committee approval

According to the Dutch law, Medical Ethical Committee approval was obtained at the principal study center. As neurologists only referred patients for the trial, the Medical Ethical Committees of just the four other regional main study centers had to approve local execution of the trial; all referring centers were given notice of this mode of conduct with a standard letter.

### Trial funding

To cover the costs of executing this investigator-initiated trial, a total of about one million Euro was obtained from The Netherlands Organization for Health Research and Development (Dutch governmental fund for health research, project number 0-82310-97-11031, approximately EUR 660,000), Stichting Parkinsonfonds, Stichting Parkinson Nederland (both Dutch charitable funding organization for PD research), and Parkinson Vereniging (Dutch patient organization). Trial medication in the first 42 weeks of the trial costed approximately EUR 250,000; a large part of other expenses went to the research nurses’ salaries. Funding for the research nurses in the five main study hospitals was provided on the basis of included patients. Thus, per included patient, there were enough resources for that hospital to perform all measurements. The contract with the pharmaceutical company providing the study medication was on the basis of a fixed contract price. Therefore, no extra funding was needed for the study duration extension.

### External assessment of recruitment process

For this goal, a version of the QuinteT Recruitment Intervention was used. A QRI is usually integrated with an RCT from the design stage to optimize recruitment, but a QRI-Two has been designed to be applied to RCTs that are underway by reviewing trial materials (protocol and patient information) alongside recruitment monitoring data [13]. In this study, the QRI-Two focused on finding issues that had inhibited recruitment that are less easily recognized—called “hidden challenges”—in addition to the “clear obstacles,” which are organizational barriers that are anticipated and known by the trial team [14, 15]. “Hidden challenges” usually relate to deep-seated issues such as clinician equipoise, strong patient preferences, or issues with the way the trial is presented [13–16]. The QRI was developed and refined over 15 years with over 60 randomized clinical trials (RCTs) with severe recruitment difficulties, and as there is observational evidence of significantly increased recruitment rates and completion of recruitment in an evaluation of five completed QRIs [17], we were interested to learn whether a retrospective review of the LEAP trial could identify hidden challenges that might have impeded recruitment. An experienced independent QRI researcher (J.L.D.) reviewed LEAP trial recruitment strategies and recruitment data.

### Data gathering

During the study, we registered data concerning all referrals and inclusion, which included the following: name and date of birth of the patient, referral date and center, referring neurologist, date of inclusion, when and which trial personnel was employed, when and which actions were undertaken aiming to improve referral or inclusion rate, and reason of non-inclusion.

### Data analyses

For the QRI-Two review, we analyzed the following. Based on the registered data, we calculated the ratio of referred patients versus possible eligible patients, based on the population at the moment the trial started including patients nationwide in January 2012. To investigate whether there were effects on referral- and inclusion rate of seasons, vacations, sending cakes at inclusion milestones, or the change of a research nurse, we visually inspected graphs showing referral and inclusion rates. Finally, we analyzed variations across centers in reasons why patients were not included. Analyses are descriptive; no statistical tests were done.

## Results

The prospective recruitment strategies mostly focused on the organizational and logistical issues, which the LEAP trial team anticipated and addressed to complete recruitment to the trial.

### Referral rate

There were 62 centers that signed the IPS. Seven academic and fifty non-academic centers participated in referring patients for the LEAP trial (Table 2); five centers eventually did not refer patients. There was a large difference in the ratio of estimated eligible patients versus referred patients of the Northwest and Southwest regions, being similar regions with respect to the composition of its population.

In 57 months, 766 patients were referred for the trial, averaging 13.4 patients per month. A lower-than-expected number of patients were referred, with large variations among regions. When analyzing the ratio of referred versus estimated eligible patients, the high ratio of 29.9% in region Southeast is of note, which we attribute to the fact that the region consisted of only a few centers that relatively referred many patients. Furthermore, the referral ratio was notably low (5.3%) in the most populous region Southwest, and high (19.3%) in region Northwest, the region of the principal study center. Both regions are similarly highly urbanized and densely populated (Table 1). Referral numbers differed widely between centers (range 1 to 86, mean 12.4 patients for the whole inclusion time per center). We could not discern a pattern in the number of referrals during seasons, vacation periods, or in the periods after we distributed cakes to participating centers. Interestingly, we did not see a change in referral rates of referring centers during the trial. Friendships between neurologists working in the principal study center and other centers did not result in a higher referral rate (data not shown).

**Table 1:** Referrals and inclusions*

Referrals and inclusions	Total	Northwest	Northeast	East	Southeast	Southwest	Unknown
Estimated population on 1/1/2012 aged 50–79 in all five regions^†^	5,284,676	1,316,362	945,173	1,295,626	211,983	1,515,531	
Estimated eligible patients during trial^‡^	6589	1641	1178	1615	264	1890	
Referred (% estimated eligible)	766 (11.6)	317 (19.3)	103 (8.7)	166 (10.3)	79 (29.9)	100 (5.3)	1
Included patients (% included of referred)	446 (58.2)	190 (59.9)	51 (49.5)	97 (58.4)	47 (59.5)	61 (61.0)	
*Not included*							
Reason (% of not included patients)							
Unknown^§^	133 (41.6)	35 (10.9)	20 (6.3)	59 (18.4)	9 (2.8)	9 (2.8)	1 (0.3)
Exclusion criterion^‖^	53 (16.6)	24 (7.5)	9 (2.8)	4 (1.3)	8 (2.5)	8 (2.5)	
Too taxing**	41 (12.8)	15 (4.7)	9 (2.8)	2 (0.6)	7 (2.2)	8 (2.5)	
Patient wanted to start with medication**	39 (12.2)	20 (6.3)	8 (2.5)	1 (0.3)	1 (0.3)	9 (2.8)	
Patient did not want medication**	35 (10.9)	24 (7.5)	1 (0.3)	2 (0.6)	3 (0.9)	5 (1.6)	
Afraid of side-effects**	6 (1.9)	3 (0.94)	3 (0.94)				
Patient could not be reached‖	4 (1.3)	2 (0.6)	1 (0.3)		1 (0.3)		
Research nurse did not receive referral**	2 (0.6)				2 (0.6)		
Participation in other clinical research‖	2 (0.6)	1 (0.3)			1 (0.3)		
Extended stay outside Netherlands^‖^	2 (0.6)	1 (0.3)		1 (0.3)			
Patient not convinced of PD-diagnosis**	1 (0.3)	1 (0.3)					
Only wants to participate if unblinded**	1 (0.3)	1 (0.3)					
Time to contact patient took too long**	1 (0.3)		1 (0.3)				
% of unknown reason relative to number of non-inclusion	41.6%	27.6%	38.5%	85.5%	28.1%	23.1%	

*The Netherlands was divided into five regions from which all regional study procedures were performed by the main study center. These were Academic Medical Center (Northwest), University Medical Center Groningen (Northeast), Radboud University Medical Center (East), Zuyderland Medical Center (Southeast), and Leiden University Medical Center (Southwest)

^†^Source: Demografische kerncijfers per gemeente 2012, ISBN: 978-90-357-2092-3

^‡^Source: reference number 9

^§^Possible partially able to include; a total of 41.6% of not included patients

^¶^One patient withdrew from the trial after inclusion but before baseline measurements and randomization and wanted its records to be destroyed

^‖^Not possible to include; a total of 19.1% of not included patients

**Patient not convinced to participate, so possibly able to include; a total of 39.4% of not included patients

### Inclusion rate

The first patient was included on 17 August 2011 and the last on 17 May 2016, amounting to a recruitment period 27 months longer than anticipated. A total of 446 patients were included (average 7.8 patients per month), which is 6.8% of the estimated eligible patients. The overall ratio of included versus referred patients was 58.2%, with only the northeast region having a somewhat lower inclusion ratio of 49.5% (Table 1). During the trial, inclusion ratios were constant for each region, with slight differences between research nurses, also noticeable when regional research nurses changed in the Northwest and East region (data not shown). The variation in the number of referrals resulted in slight variations in inclusion rate over time, as the inclusion ratio did not change during periods with lower referrals (data not shown).

Although a large number of centers indicated their intention to participate, the inclusion of patients occurred mostly in a quarter of these centers: 70.2% of the included patients were referred from only 15 centers, compared to 13 centers from which only one patient was included: 2.9% of all included patients. Eight out of the 15 centers from which the most patients were included, were situated in the region of the principal study center (region Northwest), including a total of 170 patients—38.1% of the total 446 patients (Table 2).

**Table 2:** Inclusion per type of center

	Number of centers	Included patients (% of total patients)	Mean number included patients/center
Academic center	7	111 (24.9)	15.9 (range 3–40)
Non-academic center	50	335 (75.1)	6.7 (range 1–48)
1 included patient/center	13	13 (2.9)	1
2–5 included patients/center	22	68 (15.2)	3
6–10 included patients/center	7	52 (11.7)	7
11–20 included patients/center	10	131 (29.4)	13.1
> 20 included patients/center	5	182 (40.8)	36.1
No. of centers of 15 best including centers in the main study region	8 out of 15		

### Non-inclusion

In this study, 19.1% of the referred patients that were not included turned out not to be eligible, mostly (77.4%) because before the first contact with the research nurse PD medication was already started—suggesting that there may not have been equipoise for these patients. Other reasons for non-inclusion were clearer, in that the patient could not be reached, already participated in another trial, or because of extended stays outside the Netherlands. However, there were 41.6% excluded for “unknown” reasons, which may reflect poor reporting or “hidden” issues with eligibility/equipoise [15]. A further 39.4% of the reasons for non-inclusion were related to the patients’ wishes, for example, wanting to be included in the placebo or treatment arm of the study, fear of the study being too taxing, or fear of side effects (Table 1). Further, although numbers are small, in the Northeast and Southwest region, more patients were excluded because they had either started or wanted medication compared with those who did not want medication (Table 1).

### “Hidden” challenges

The review of recruitment and monitoring data found some indications of potential issues with eligibility, equipoise, and study presentation. These included variations in the contributions of centers to enrollment even when populations were similar, the reporting of patient preferences for one treatment or another (not the trial) among 39% of potential participants, and patients not eligible for the trial because they had already been started on medication. The LEAP trial team reported that while the topic of the trial was of great clinical interest, and there was sufficient “community equipoise” to launch the trial, contemporaneous notes and monitoring data indicated that some centers favored treatment with medication and others with delaying medication either when they joined or during recruitment. Together, these are all clues to potential issues with equipoise, eligibility, and managing patient preferences in some centers or among some clinicians [14].

## Discussion

The LEAP trial was a large, successful, investigator-initiated landmark medication trial answering a burning clinical question on a limited budget. It showed that in early Parkinson’s disease, levodopa/carbidopa 100/25 mg TID had no disease-modifying effect over 80 weeks [7]. However, it did take more than 2 years longer than planned to deliver recruitment.

The clinical relevance of the trial and the minimal effort required meant that general neurologists throughout the Netherlands could be enthused to take part, which was reflected in the high number of centers participating in the trial. Because general neurologists usually tend not to participate in trials, we mark their participation and the formation of an informal research network as a key spin-off achievement of the LEAP trial. The LEAP team developed and implemented a large number of strategies to optimize recruitment, including (a) efficiently streamlining trial procedures such as centralizing medication provision and Medical Ethical Committee-approval, (b) raising trial awareness among neurologists, (c) ensuring trial processes were easy to complete and took as little time as possible, (d) supporting centers when they started to recruit with frequent communication and rewards for success, and (e) ensuring participation was easy and as pleasant as possible for patients by liaising with patient groups and having home visits and continuity of nursing staff. The administrative burden was diminished greatly by only having to obtain Medical Ethical Committee approval in the five main study centers, while patients were being referred from 57 centers, and because all trial medication-related tasks were outsourced to one central party. Therefore, the research team in the principal study center could be kept small and focused on trying to improve recruitment and inclusion rate and maintaining an up-to-date trial administration. The combination of all these factors resulted in the low costs for this large trial, including the cost-neutral extension of the trial. Funding for the research nurses was provided per included patient, and as recruitment lagged, research nurses were seldom occupied with the trial on a full-time basis, and also had some other work in their respective hospitals. The contract with the pharmaceutical company that provided the study medication was on the basis of a fixed contract price; therefore, no extra funding was needed for the trial extension.

As the referral rate was considerably lower than expected, the extended recruiting period has been partly compensated by the high inclusion ratio of 58.2% of the referred patients. One of the key contributors of the high inclusion ratio may have been the combination of optimal training of research nurses, ample time for face-to-face contact for the patient with mostly the same research nurse, and the convenience that assessments could take place at the location of the patients’ preference [18]. Although the LEAP team implemented many different strategies to improve the referral and inclusion rate, it remains unclear what the contribution to the inclusion rate was for each intervention. For an investigator-initiated trial, every spent hour counts heavily as the budget is usually limited. It would be very helpful to know which strategies in different study contexts work best to raise referral and inclusion rates; if more funding would have been available, we could, for example, have hired more supporting staff to further pursue these strategies.'

Despite the fact that the LEAP trial completed its inclusion goal, it took over 2 years longer than expected, even though a large number of centers participated in the study. Reviewing the recruitment data, we noted that a relatively small number (15) of the participating centers referred the majority of patients (70.2%). In contrast, a similar number—13 centers—only referred one patient. The inclusion criteria required that patients had to be referred shortly after being diagnosed with PD—often an emotional moment, which could have influenced the willingness for a patient or neurologist to discuss referral for a study—suggesting the possible involvement of a “hidden challenge” such as equipoise or issues with study presentation in some of the centers. The aimed for inclusion ratio of all estimated eligible patients of about 20% proved to be too ambitious, although prior to the study, we did not possess data to have a more realistic estimation. It is a key priority for future investigator-initiated trials to have better data to estimate recruitment rates [19].

In this context, the steady referral rate of all participating centers throughout the trial is of interest, particularly despite the efforts to improve it—as this also suggests that there may have been other factors at work. Trial teams need to decide whether to focus resources on improving inclusion ratios or referral rate to enable as many eligible patients as possible to consider participation [13].

Furthermore, as a majority of the centers from which the most patients were included were situated in the region of the principal study center, this shows that this is apparently a factor that boosts the willingness to participate in referring patients. The large difference in the ratio of estimated eligible patients versus referred patients of the Northwest and Southwest regions supports this opinion. These centers had interns, residents, or neurologists previously trained at the principal study center, suggesting that these centers could have a higher commitment to the trial. Previous QRIs also suggest such variations might also signal that sites varied in their assessment of eligibility and/or equipoise [19, 20].

In our trial, 39.4% of the not included patients did not participate because of reasons related to the patients’ opinion, possibly leaving room for improvement of the inclusion ratio. The presence of patient preferences distributed across the interventions is often an indicator of underlying difficulties with equipoise that are reflected in the way the trial is presented to patients [21]. Finally, the fact that in the Northeast and Southwest region many more patients were excluded because they had either started or wanted medication compared with those who did not want medication, could further reflect issues with equipoise or study presentation noted above [14, 16, 21].

Since QRI research has shown that clinicians who are more comfortable with equipoise can present the study more effectively, a QRI integrated prospectively would have investigated the reasons of neurologists to refer patients and the way they and the research nurses presented the trial to patients [16]. Without recordings, it is unknown whether this issue played a role in the LEAP trial, but it could help to understand why there were differences in referral and inclusion rates. If a QRI had been integrated into the LEAP trial, more comprehensive data would have been collected about patient screening, eligibility assessment, whether patients were approached, and then accepted or declined randomization—using the Screened, Eligible, Approached Randomised (SEAR)-framework [19]. This would have provided evidence about the issues that might have inhibited recruitment, helping find strategies to improve referral and inclusion rates and reducing any center variations [13]. The QRI-Two review was limited by being undertaken after completion of recruitment.

This study has shown that the LEAP trial—an investigator-initiated medication trial studying a neurodegenerative disease in an early stage—was successful in achieving the aim for sample size. This was achieved on a limited budget by focusing on minimizing administrative and organizational procedures. However, the trial did take 2 years longer than anticipated to complete recruitment. A review of recruitment processes and monitoring data showed that centers with closer institutional ties to a principal study center tended to have a higher referral rate, and variations in referrals and reasons of non-inclusion suggested the presence of “hidden” challenges related to clinical equipoise, patient eligibility, or study presentation. Integrating a recruitment intervention such as a QRI prospectively could have explored the hidden challenges and found solutions that could have increased recruitment efficiency.

## Data Availability

For academic research, de-intentified data used for analyses presented in this article can be requested from the corresponding author.
